# Metabolic pathway engineering using the central signal processor P_II_

**DOI:** 10.1186/s12934-015-0384-4

**Published:** 2015-11-25

**Authors:** Björn Watzer, Alicia Engelbrecht, Waldemar Hauf, Mark Stahl, Iris Maldener, Karl Forchhammer

**Affiliations:** Interfaculty Institute of Microbiology and Infection Medicine Tübingen, Eberhard-Karls-Universität Tübingen, Auf der Morgenstelle 28, 72076 Tübingen, Germany; Central Facilities, Analytics, ZMBP, Eberhard-Karls-Universität Tübingen, Auf der Morgenstelle 32, 72076 Tübingen, Germany

**Keywords:** Cyanophycin, Cyanobacteria, l-Arginine, P_II_ protein

## Abstract

**Background:**

P_II_ signal processor proteins are wide spread in prokaryotes and plants where they control a multitude of anabolic reactions. Efficient overproduction of metabolites requires relaxing the tight cellular control circuits. Here we demonstrate that a single point mutation in the P_II_ signaling protein from the cyanobacterium *Synechocystis* sp. PCC 6803 is sufficient to unlock the arginine pathway causing over accumulation of the biopolymer cyanophycin (multi-l-arginyl-poly-l-aspartate). This product is of biotechnological interest as a source of amino acids and polyaspartic acid. This work exemplifies a novel approach of pathway engineering by designing custom-tailored P_II_ signaling proteins. Here, the engineered *Synechocystis* sp. PCC6803 strain with a P_II_-I86N mutation over-accumulated arginine through constitutive activation of the key enzyme *N*-acetylglutamate kinase (NAGK).

**Results:**

In the engineered strain BW86, in vivo NAGK activity was strongly increased and led to a more than tenfold higher arginine content than in the wild-type. As a consequence, strain BW86 accumulated up to 57 % cyanophycin per cell dry mass under the tested conditions, which is the highest yield of cyanophycin reported to date. Strain BW86 produced cyanophycin in a molecular mass range of 25 to >100 kDa; the wild-type produced the polymer in a range of 30 to >100 kDa.

**Conclusions:**

The high yield and high molecular mass of cyanophycin produced by strain BW86 along with the low nutrient requirements of cyanobacteria make it a promising means for the biotechnological production of cyanophycin. This study furthermore demonstrates the feasibility of metabolic pathway engineering using the P_II_ signaling protein, which occurs in numerous bacterial species.

**Electronic supplementary material:**

The online version of this article (doi:10.1186/s12934-015-0384-4) contains supplementary material, which is available to authorized users.

## Background

Cyanophycin (multi-l-arginyl-poly-l-aspartate) is a nitrogen/carbon reserve polymer present in most cyanobacteria [1, 2] and in a few heterotrophic bacteria [3, 4]. It consists of a polyaspartic backbone with arginine residues linked via isopeptide bonds at the free carboxylate groups of the aspartic backbone [5, 6]. Cyanophycin is nonribosomally synthesized from arginine and aspartate by cyanophycinsynthetase (CphA) in an ATP-dependent elongation reaction using unidentified primers [6–8]. Cyanophycin accumulates in the cytoplasmic space as opaque, membrane-less granules [9]. Isolated cyanophycin has a molecular weight widely ranging from 25 to 100 kDa [5] and is insoluble at physiological pH, but soluble in diluted acids or bases [10].

In cyanobacteria, the amount of cyanophycin is usually less than 1 % of the cell dry mass during exponential growth. When cells experience certain unfavorable conditions other than nitrogen starvation, such as stationary phase or unbalanced growth conditions owing to nutrient limitation, e.g., sulfate or phosphate starvation [11], light stress, low temperature [12], or presence of chloramphenicol [13], the cyanophycin content may increase to 18 % of the cell dry mass. Cyanophycin also can accumulate transiently during the recovery of nitrogen-starved, non-diazotrophic cyanobacteria upon addition of a usable nitrogen source [14]. Furthermore, in heterocysts (specialized cells for nitrogen fixation) of cyanobacteria of the order *Nostocales,* polar nodules consisting of cyanophycin are deposited at the contact site to adjacent vegetative cells [15].

The cyanobacterial P_II_ protein is a member of the widely distributed family of P_II_ signal transduction proteins present in bacteria, plants, and some archaea [16]. P_II_ proteins are largely involved in the regulation of nitrogen assimilatory metabolism. For this purpose, P_II_ senses the cellular energy level by binding ATP or ADP competitively [17] and senses the state of central carbon/nitrogen metabolism by binding or lack of binding of the status reporter molecule 2-oxoglutarate (2-OG) [18, 19]. Effector molecule binding results in structural rearrangements of the large surface-exposed T-loops of P_II_, its major protein-interaction determinant. In the unicellular freshwater cyanobacteria *Synechococcus elongatus* PCC 7942 and *Synechocystis* sp. PCC 6803 during nitrogen starvation, which corresponds with high 2-OG levels, the P_II_ protein binds 2-OG and is phosphorylated at the apex of the T-loop at position Ser49; when nitrogen is in excess, which corresponds to 2-OG paucity and therefore no binding of the P_II_ protein to 2-OG, Ser49 is dephosphorylated [16, 20].

Depending on the bound effector molecules and the phosphorylation status, P_II_ interacts and influences many target proteins, including enzymes, channels, and regulatory proteins [18, 21, 22]. One of the major P_II_ target proteins is the enzyme *N*-acetylglutamate kinase (NAGK) [23], which catalyzes the committed step in the cyclic arginine biosynthesis pathway [24]. Under nitrogen excess, P_II_ in its non-phosphorylated form binds to NAGK [25], there by strongly enhancing its biosynthetic activity as well as relieving the feedback inhibitory effect of arginine on NAGK activity [18]. In a screening for P_II_ variants with altered NAGK binding properties, our laboratory previously identified a variant of the *S. elongatus* PCC 7942 P_II_ protein with a single amino acid replacement, Ile86 to Asp86, hereafter referred to as P_II_(I86N), that constitutively binds NAGK in vitro [26]. The variant is a structural mimic of P_II_ in the NAGK complex, with its T-loops in a kinked conformation; as a consequence of this special T-loop folding, this variant has a high affinity for NAGK and no longer responds to 2-OG but can bind citrate in vitro [27].

**Fig. 1:**
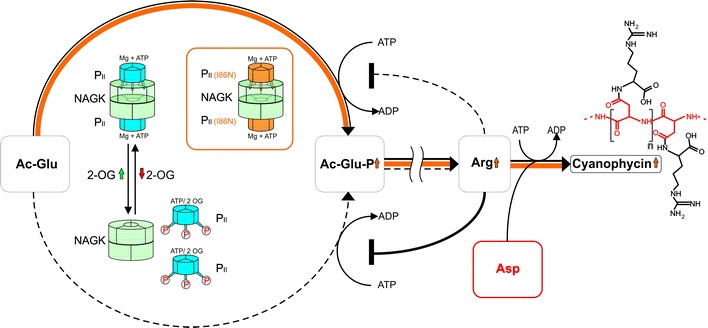
The strategy of metabolic engineering of the P_II_(I86N) protein in *Synechocystis* sp. PCC 6803 for arginine/cyanophycin overproduction. The conversion of *N*-acetylglutamate (Ac-Glu) to *N*-acetylglutamate-phosphate (Ac-Glu-P) is the rate-limiting step in the cyclic arginine synthesis pathway and is catalyzed by NAGK. NAGK activity is controlled by complex formation with the P_II_ protein, which senses the nitrogen status by 2-oxogluterate (2-OG) binding. In the wild-type, unbound NAGK has low activity (*dashed line*) and is highly susceptible to arginine feedback inhibition, whereas NAGK bound to the P_II_ protein has high activity (*solid black line*) and much less sensitive towards arginine. The P_II_(I86N) variant of strain BW86 (in *orange box*) permanently binds to NAGK and strongly increases its activity (*solid orange line*) and relieves arginine feedback inhibition. When excess arginine (Arg) is produced cyanophycin is synthesized from arginine and aspartate (Asp in *red*)

For in vivo studies of the role of P_II_ in arginine metabolism, strain *Synechocystis* sp. PCC 6803 offers the advantage over *S. elongatus* that it produces cyanophycin. In a P_II_-deficient mutant of *Synechocystis* sp. PCC 6803, not only does NAGK remain in a low activity state, but also the transient accumulation of cyanophycin that normally occurs after exposing a nitrogen-starved culture to excess ammonia is impaired [23]. We tested whether the opposite phenotype in *Synechocystis* sp. PCC 6803 would be possible if we replaced the wild-type *glnB* gene (encoding P_II_) with a *glnB* variant with codon alteration Ile86 to Asp, thereby generating a P_II_ variant that constitutively activates NAGK, which could lead to the accumulation of cyanophycin not just transiently, but in high amounts. This metabolic pathway engineering via manipulation of the P_II_ signal indeed resulted in a strain that excessively overproduces cyanophycin (Fig. 1).

## Results and discussion

### Expression of the P_II_(I86N) variant in *Synechocystis* sp. PCC 6803 causes a strong in vivo activation of NAGK

Previous biochemical studies have shown that the P_II_(I86N) variant of *S. elongatus* PCC 7942 constitutively binds to NAGK in vitro [26]. To test whether this P_II_ variant affects the in vivo activity of NAGK, we constructed a genomic mutant of *Synechocystis* sp. PCC 6803 in which the *glnB* gene was replaced by a *glnB* gene carrying the mutation for I86N together with a spectinomycin resistance cassette via homologous recombination. Complete segregation of the mutation in the polyploidy *Synechocystis* sp. strain, named strain BW86, was confirmed via PCR (Additional file 1: Figure S1).

**Fig. 2:**
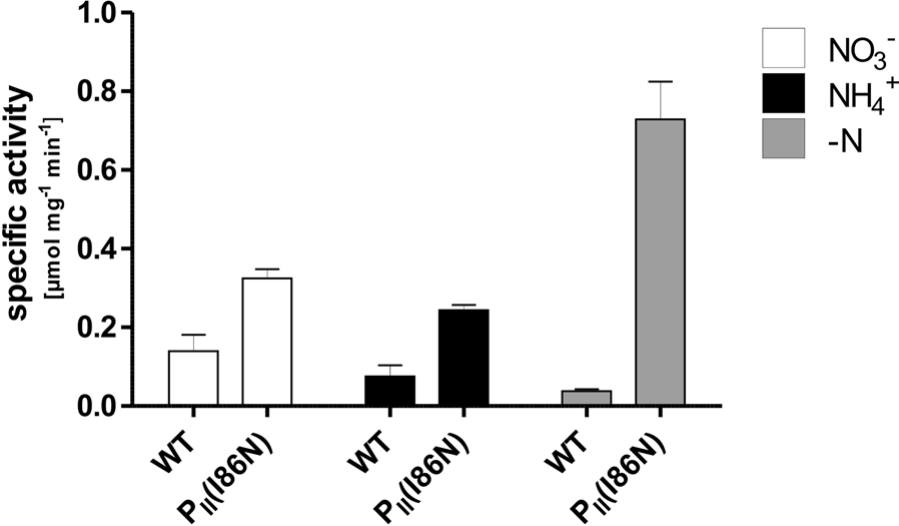
NAGK specific activity in extracts of *Synechocystis* sp. PCC 6803 (wild-type) and the engineered strain BW86 incubated with nitrate (NO_3_^−^), ammonium (NH_4_^+^), or no nitrogen source (–N) until the late exponential phase (OD_750_ of about 0.8). For nitrogen starvation (–N), cells were incubated in nitrate-supplemented BG–11 medium to an OD_750_ of 0.5 and were then transferred to BG-11 medium without a nitrogen source and incubated for 2 days to an OD_750_ of 0.8

To determine in vivo NAGK activity during growth with different nitrogen sources, we cultivated the wild-type *Synechocystis* sp. PCC 6803 and strain BW86 in BG-11 medium containing either nitrate, ammonia, or no nitrogen source (Fig. 2). NAGK activity was higher in strain BW86 than in the wild-type in all cases; the activity was 2.3-fold higher after growth with nitrate and 3.2-fold higher after growth with ammonium. The nitrogen source, i.e., nitrate or ammonium, did not strongly affect NAGK activity. However, under nitrogen starvation, NAGK activity strongly increased in strain BW86 and decreased in the wild-type, such that the activity was 19.2-fold higher in strain BW86. The low activity of NAGK in the wild-type under nitrogen starvation could be explained by the full phosphorylation of P_II_ under these conditions [28] since this prevents P_II_-NAGK interaction and thus, the NAGK enzyme would be in an inactive state [25]. Transcriptome studies of *Synechocystis* show an induced expression of the *glnB* and *argB* (encoding NAGK) genes under nitrogen starvation [29]. Such an induced expression would lead to increased levels of P_II_(I86N) and NAGK. Provided that phosphorylation of P_II_(I86N) is impaired, this could be the cause of the high NAGK activity shown in Fig. 2, since P_II_ in its non-phosphorylated state interacts with NAGK and strongly enhances its activity [25].

### The P_II_(I86N) variant has a reduced phosphorylation

**Fig. 3:**
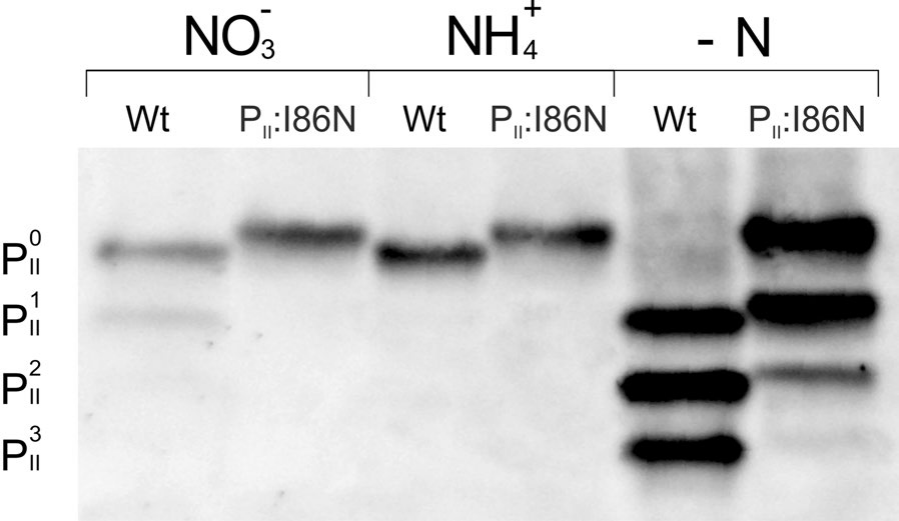
Analyses of P_II_ phosphorylation of wild-type P_II_ and P_II_(I86N) from cells grown on nitrate, ammonium, or without a nitrogen source. Native PAGE and western blotting with anti-P_II_ antibodies. 10 µg of protein crude extract per lane

To test the assumption that the high activity of NAGK in strain BW86 is due to impaired phosphorylation of P_II_(I86N), we analyzed its phosphorylation status using non-denaturing PAGE followed by immunoblotting using P_II_ specific antibodies [30] (Fig. 3). Each phosphorylation event increases the negative charge of the P_II_ protein and, therefore, leads to three isoforms of increasing electrophoretic mobility corresponding to one- two- and threefold phosphorylated forms (P_II_^1^, P_II_^2^, P_II_^3^). The mobility of non-phosphorylated P_II_(I86N) and wild-type P_II_ slightly differed due to the replacement of isoleucine with asparagine at position 86. In nitrate-grown cells, wild-type P_II_ was in the non-phosphorylated state (P_II_^0^) and in the mono-phosphorylated state (P_II_^1^), whereas P_II_(I86N) was only in the non-phosphorylated state. As expected, both P_II_ proteins were non-phosphorylated when the cells were grown with ammonium as nitrogen source. Under conditions of nitrogen starvation, P_II_ in wild-type cells was strongly phosphorylated with the two-and three-fold phosphorylated forms (P_II_^2^, P_II_^3^) prevailing. By contrast, phosphorylation of P_II_(I86N) was severely impaired, with absence of the fully phosphorylated form P_II_^3^ but dominance of the non-phosphorylated (P_II_^0^) and mono-phosphorylated form of P_II_. Furthermore, due to the induced expression of *glnB* during nitrogen starvation [21], the bands of wild-type P_II_ and P_II_(I86N) from nitrogen-starved cell are more intensive compared to non-starved cells (Fig. 3). These data confirm that the strikingly high activity of NAGK under nitrogen starvation in strain BW86 (Fig. 2) is due to impaired phosphorylation of P_II_(I86N).

### The high NAGK activity of the P_II_(I86N) variant leads to a high intracellular arginine level

**Fig. 4:**
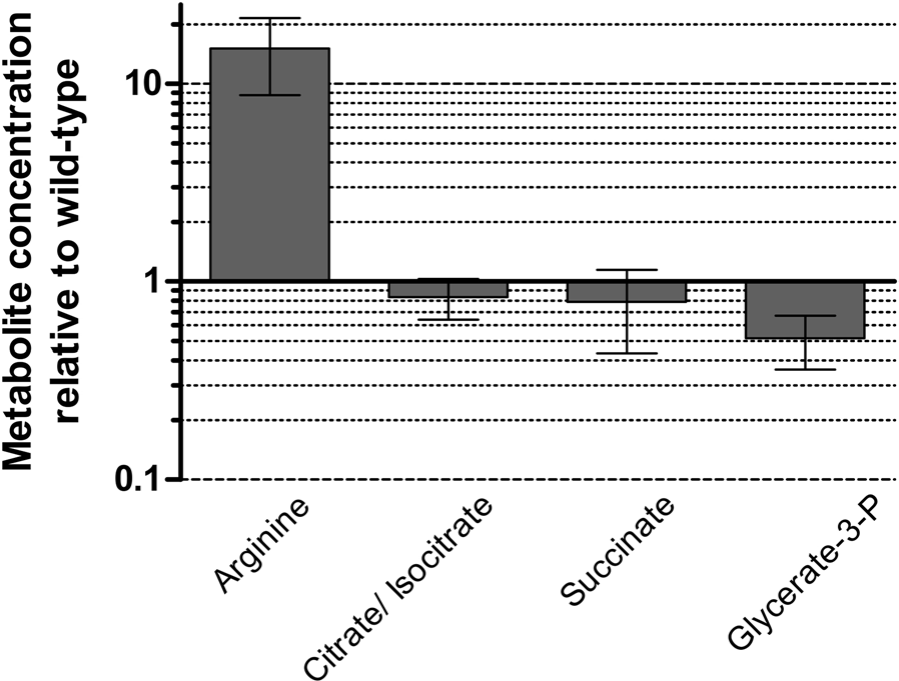
Concentration of metabolites of strain BW86 relative to that of wild-type *Synechocystis* sp. PCC 6803. Only those metabolites are shown whose levels in the two strains differed. Cells were grown in BG-11 medium with nitrate to an OD_750_ of 0.8; n = four independent replicates. The method does not distinguish between citrate and isocitrate. Note that the x-fold differences in concentration are on a log 10 scale

NAGK represents a key enzyme in the regulation of arginine biosynthesis and as such its activity is feedback-controlled by the cellular arginine levels, the final product of the pathway. Importantly, in vitro analysis demonstrated that complex formation of NAGK with P_II_ not only activates the enzyme, but strongly relieves feedback-inhibition by arginine [26]. To reveal the metabolic changes that are caused by the replacement of wild-type P_II_ by the P_II_(I86N) variant in vivo, we determined the metabolome of the two strains with a focus on metabolites of primary metabolism, i.e., of the TCA cycle, CO_2_ fixation, amino acid biosynthesis, and glycolysis (Fig. 4). For the comparison an untargeted metabolomics approach was chosen. According to PCA and OPLS-DA analysis (see “Methods”), the only changing metabolites are arginine, citrate/isocitrate, succinate and glycerate-3-P. Remarkably, strain BW86 accumulated on average 15-fold more arginine than the wild-type. This increase reflects the constant activation of NAGK by the P_II_(I86N) variant, which maintains NAGK in a state that is highly insensitive towards arginine-feedback inhibition. On the other hand, the pools of citrate/isocitrate, succinate and glycerate-3-P were decreased. The pools of citrate/isocitrate and succinate were just slightly (p value >0.05) lower in strain BW86 whereas the amount of glycerate-3-P was significantly lower in strain BW86 (p value 0.0107). The reason for the decreased level of glycerate-3-P is not known, but it indicates a yet to be explored connection to the increased metabolite flow into the arginine pool, e.g. by accelerated glycolytic flux that could drain the glycerate-3-P pool.

### The P_II_(I86N) mutation leads to cyanophycin accumulation

**Fig. 5:**
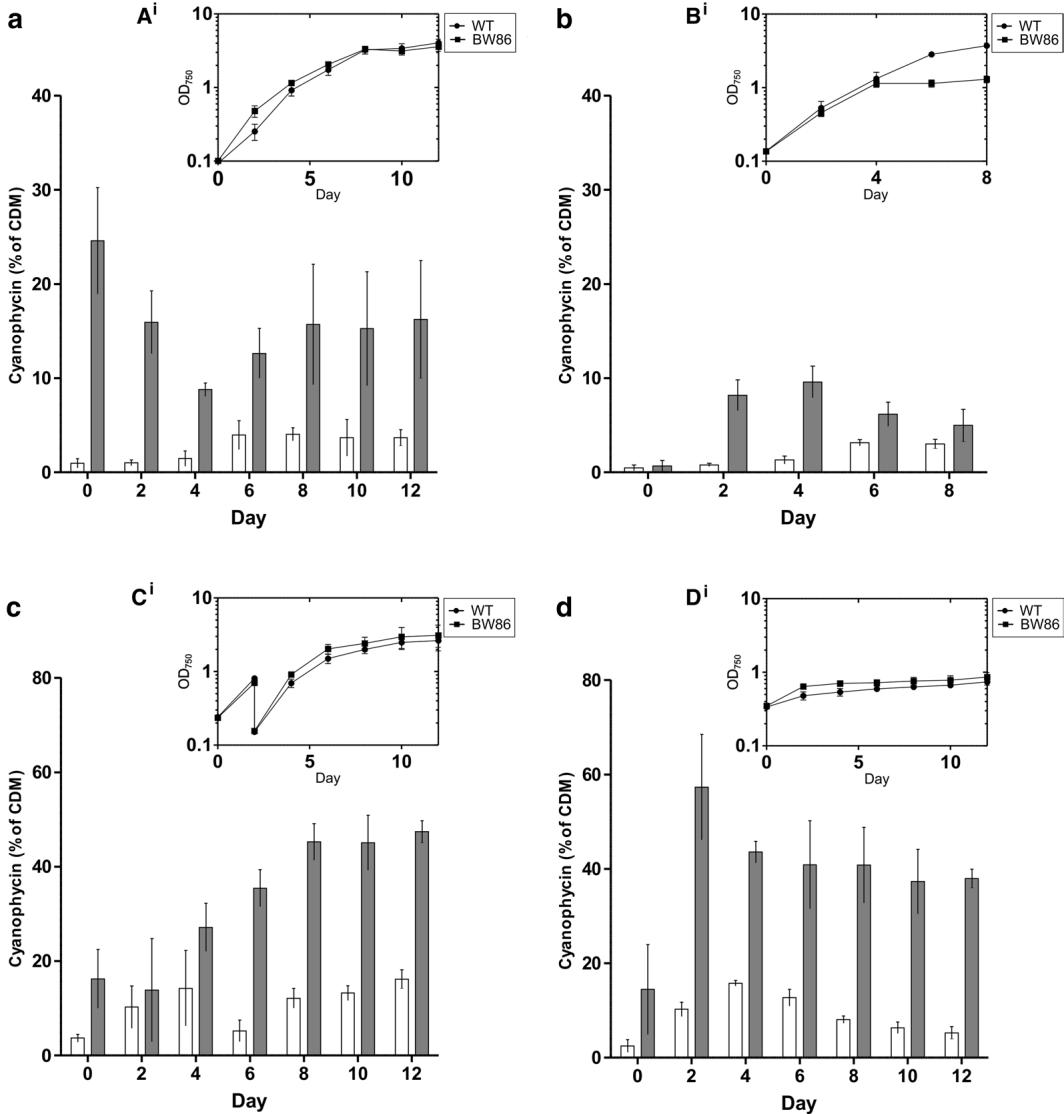
Cyanophycin accumulation in *Synechocystis* sp. PCC 6803 wild-type (*white bars*) and strain BW86 (*gray bars*) cultivated **a** with nitrate, **b** with ammonium, **c** under phosphate starvation, and **d** under potassium starvation. *A* ^*i*^–*D* ^*i*^ Growth curve of *Synechocystis* sp. PCC 6803 (WT) (*circles*) and strain BW86 (*squares*). Y -axes in log 10 shows the OD_750_. Cells of each strain exponentially growing in BG-11 medium were inoculated into 800 ml of the respective BG-11 medium and incubated with an influx of 2 % CO_2_ in air. For phosphate starvation, washed cells were grown in phosphate-free medium and were diluted with fresh phosphate-free medium after 2 days of cultivation; this time point was day 2. For potassium starvation, the cells of the inoculum were washed and inoculated into potassium-free medium. In **c** and **d**, the nitrogen source was nitrate. Cyanophycin was quantified every second day; the concentration is plotted against the cell dry mass (CDM). Note the different scales on the y-axes

Previous studies have shown a relationship between a lack of P_II_-dependent NAGK activation and a lack of cyanophycin accumulation. It has been suggested that the lack of P_II_-induced arginine accumulation disables the accumulation of cyanophycin [23]. As shown above, strain BW86 displays enhanced NAGK activity due to constant NAGK activation by the P_II_(I86N) variant and in consequence, over produces arginine. It was, therefore, intriguing to elucidate how this affects the accumulation of cyanophycin. Preliminary analysis indicated, that indeed the cellular cyanophycin content was strongly increased. Next, we systematically determined the relationship between nutritional conditions and cyanophycin production (Fig. 5). With nitrate as nitrogen source (Fig. 5a), wild-type cells accumulated about 1.1 ± 0.5 % cyanophycin relative to the cell dry mass (CDM) in the first 4 days. As the cells entered stationary phase on day 6 up to day 12 (Fig. 5A^i^), cyanophycin slightly increased up to 3.6 ± 0.8 % of the CDM. By contrast, *s*train BW86 accumulated up to 15.6 ± 5.4 % cyanophycin relative to the CDM, i.e., on average almost six fold more than the wild-type. Remarkably, upon inoculation of a fresh BW86 culture with stationary cells, the cyanophycin content was initially very high, but the level transiently decreased in the following days, corresponding to the exponential growth phase. After entry into stationary phase, the cyanophycin content increased again.

With ammonium as nitrogen source (Fig. 5b), wild-type cells accumulated cyanophycin similarly as with nitrate as nitrogen source, whereas strain BW86 produced less cyanophycin than in nitrate-supplemented medium, but still considerably more than the wild-type. The cyanophycin level increased up to 9.6 ± 1.7 % of the CDM in the first 4 days but began to slightly decrease on day 6. Quantification of the amount of ammonium in the medium supernatant of the two strains indicated that strain BW86 consumed ammonia more slowly than the wild-type (Additional file 2: Figure S2). In the first 2 days of cultivation, ammonia consumption of the wild-type and the BW86 strain was similar (Additional file 2: Figure S2). Subsequently, ammonia consumption in strain BW86 ceased. It is conceivable that the initial uptake of ammonium allowed initial cyanophycin accumulation, resulting in a peak of cyanophycin amount at day 2 and 4 (Fig. 5b). However, since after 4 days the strain BW86 stopped to consume ammonia and to grow (Fig. 5B^i^), the cells might have degraded their cyanophycin reserves and used them as internal nitrogen source. The impaired ammonia consumption of strain BW86 could indicate a possible role of P_II_ in ammonium utilization, which might be affected in the P_II_(I86N) variant. In *Escherichia coli* and many other heterotrophic bacteria, the P_II_ homologue GlnK regulates the membrane-localized ammonia transporter AmtB [18]. Although an involvement of cyanobacterial P_II_ in the regulation of Amt homologues has not yet been clearly demonstrated, such a function seems possible [28]. The phenotype of impaired ammonia utilization observed here could indicate a direct involvement of P_II_ in ammonia uptake in *Synechocystis* sp. as well.

As nitrate-grown cells had higher cyanophycin contents than ammonium-grown cells, we used nitrate as the nitrogen source in the following studies. In some cyanobacterial species, the cyanophycin content can increase up to 18 % of the CDM [11–13, 31] under certain stress-conditions. Generally, cyanophycin accumulation is triggered by conditions of reduced growth rate, such as entry into stationary phase or unbalanced cultivation conditions, whereas during exponential growth, the amino acids arginine and aspartate are mostly used for protein biosynthesis. To test the effect of growth limitation on cyanophycin accumulation, we starved cells for phosphate or potassium (Fig. 5c, d, respectively).

To induce phosphate starvation cells were washed and inoculated in phosphate free BG-11 medium. Since the internal phosphate pools are only slowly depleted, the cell growth in phosphate free medium is initially not affected. In order to avoid growth into stationary phase without full induction of phosphate starvation, after 2 days, the cultures were again diluted 1:5 in phosphate free medium. After two more days, growth ceases due to phosphate starvation. Accordingly, the cyanophycin content of both strains on day 0 and day 2 (Fig. 5c) is comparable to that of non-starved cells (Fig. 5a). In agreement, the growth rate at this early stage was not affected because of a sufficient internal phosphate pool (Fig. 5C^i^) [32]. On day 4, the cyanophycin content in both the wild-type and strain BW86 strongly increased, correlating to the onset of the phosphate starvation. After 12 days, the accumulation of cyanophycin was maximal, with the wild-type exhibiting 16.2 ± 1.9 % of the CDM and strain BW86 47.4 ± 2.3 % of the CDM.

Potassium starvation is a very strong and immediate stress for cyanobacterial cells [33] because it is not buffered by internal pools and stringently affects the growth. In the absence of potassium, the growth rate of both strains was nearly zero [33] (Fig. 5D^i^). To test the effect of potassium starvation on cyanophycin production, exponentially growing cells of both strains were washed and inoculated into potassium-free BG-11 medium (Fig. 5d). In contrast to phosphate starvation, the cyanophycin content in potassium-starved cells rapidly increased in the first 2 days, due to the rapid arrest of growth. As long as the metabolism is in an active state, growth arrest allows the efficient synthesis of reserve materials. In agreement, cyanophycin accumulation in the wild-type reached a peak of 15.8 ± 0.5 % of the CDM after 4 days. In strain BW86, a peak of 57.3 ± 11.1 % cyanophycin per CDM was already reached on day 2. Thereafter, the cyanophycin content of both strains slowly decreased, probably a consequence of a stress response in the decaying cells, caused by the harmful lack of potassium.

### Strain BW86 produces cyanophycin of high molecular mass

**Fig. 6:**
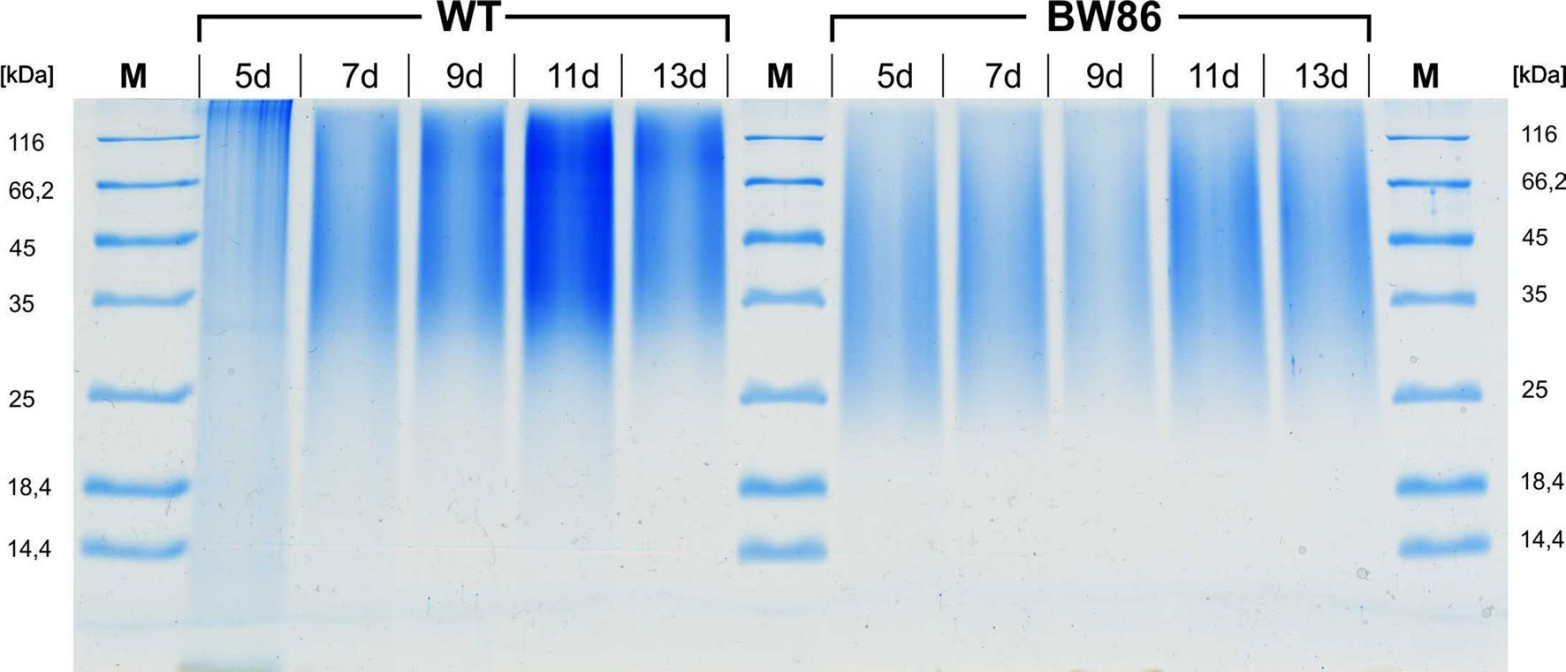
SDS-PAGE determination of the molecular weight of cyanophycin isolated from *Synechocystis* sp. PCC 6803 wild-type (WT) and strain BW86 at the indicated time points (in days) from phosphate-starved cultures. Per lane, 40 µg cyanophycin was loaded

To test the influence of the P_II_(I86N) mutation on the cyanophycin polymer length, we isolated cyanophycin granules from cells during the course of a phosphate starvation experiment. We used an extraction method that prevents hydrolyzation of the polymer that normally occurs during the usual acid extraction protocol (see “Methods”). The isolated polymer was solubilized in SDS sample buffer and analyzed by SDS-PAGE (Fig. 6). Cyanophycin isolated from strain BW86 had a size range of 25 to well above 100 kDa; the lowest molecular mass was slightly lower than that of the wild-type (range 30 to >100 kDa). Like in the wild-type, the time point at which samples were taken had no influence on the size distribution of the polymer. The high molecular weight of cyanophycin produced in strain BW86 is one of the main differences to other recombinant cyanophycin productions strains using heterologous expression systems with heterotrophic bacteria or genetically engineered yeast or plants harboring a cyanobacterial cyanophycin synthetase gene [34–36]. Those strains produce cyanophycin with a size range of only 25–45 kDa, but why they fail to produce higher molecular mass cyanophycin has not been elucidated so far. A possible explanation would be that cyanophycin synthesis in the native *Synechocystis* PCC 6803 background (as is provided in the presently engineered strain BW86) involves additional factors contributing to the polymer length. Further work on the molecular biology of cyanophycin granule synthesis is required to solve this question.

### Microscopic examination of cyanophycin production in strain BW86

**Fig. 7:**
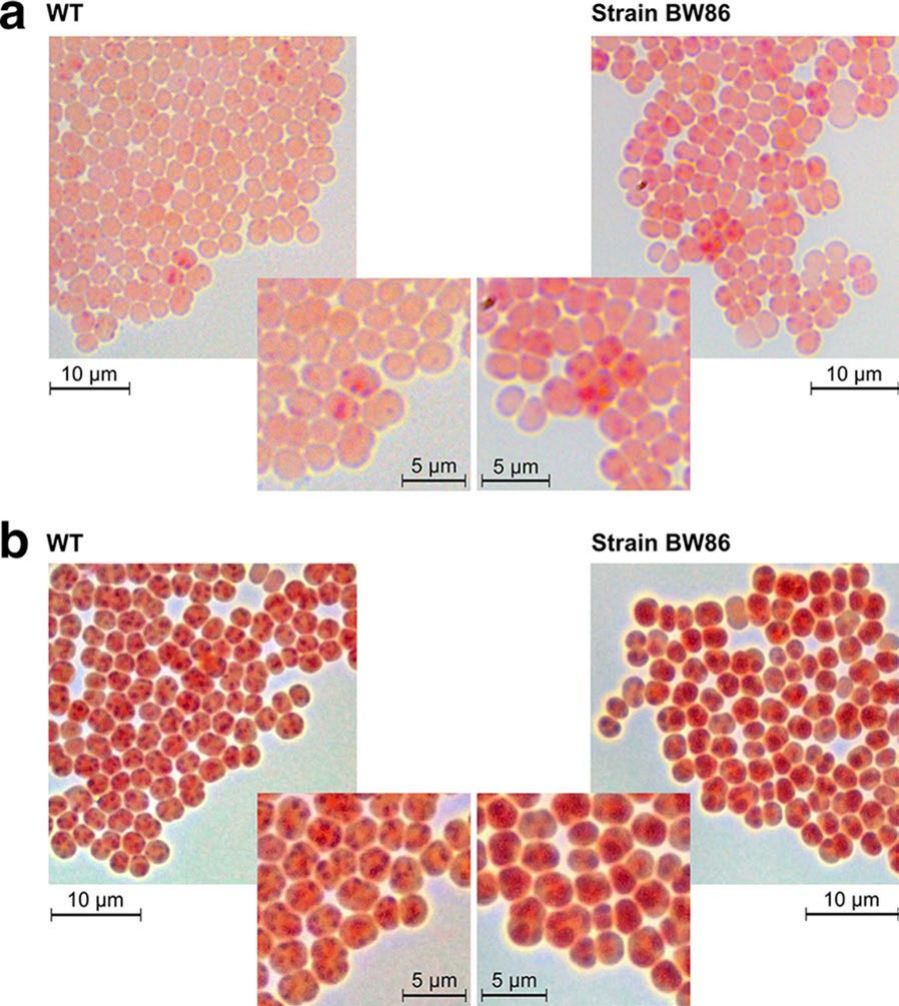
Cyanophycin stained in **a** exponentially growing and **b** phosphate-starved cells of *Synechocystis* sp. PCC 6803 wild-type and strain BW86 using the Sakaguchi reaction. The intensity of *red* indicates the amount of arginine. The dark *red* to *purple dots* are cyanophycin granules

The above experiments showed that cells of strain BW86 massively overproduced cyanophycin during exponential growth and even more, under growth-limited conditions, whereas wild-type cells under both conditions produced very little amounts of cyanophycin. To gain insight into the distribution of cyanophycin production in the population of cells of strain BW86, we attempted to microscopically visualize cyanophycin. In previous studies, cyanophycin granules have been recognized by their appearance as light dense granules in bright-field images. Consequently, clear identification of granules is difficult and only large granules could be recognized. The Sakaguchi reaction is a colorimetric reaction for identifying and quantifying arginine [37]. To use the Sakaguchi reaction in unicellular cyanobacteria, we developed a fixation protocol that maintained the structure of cells and cyanophycin granules (Fig. 7).

**Fig. 8:**
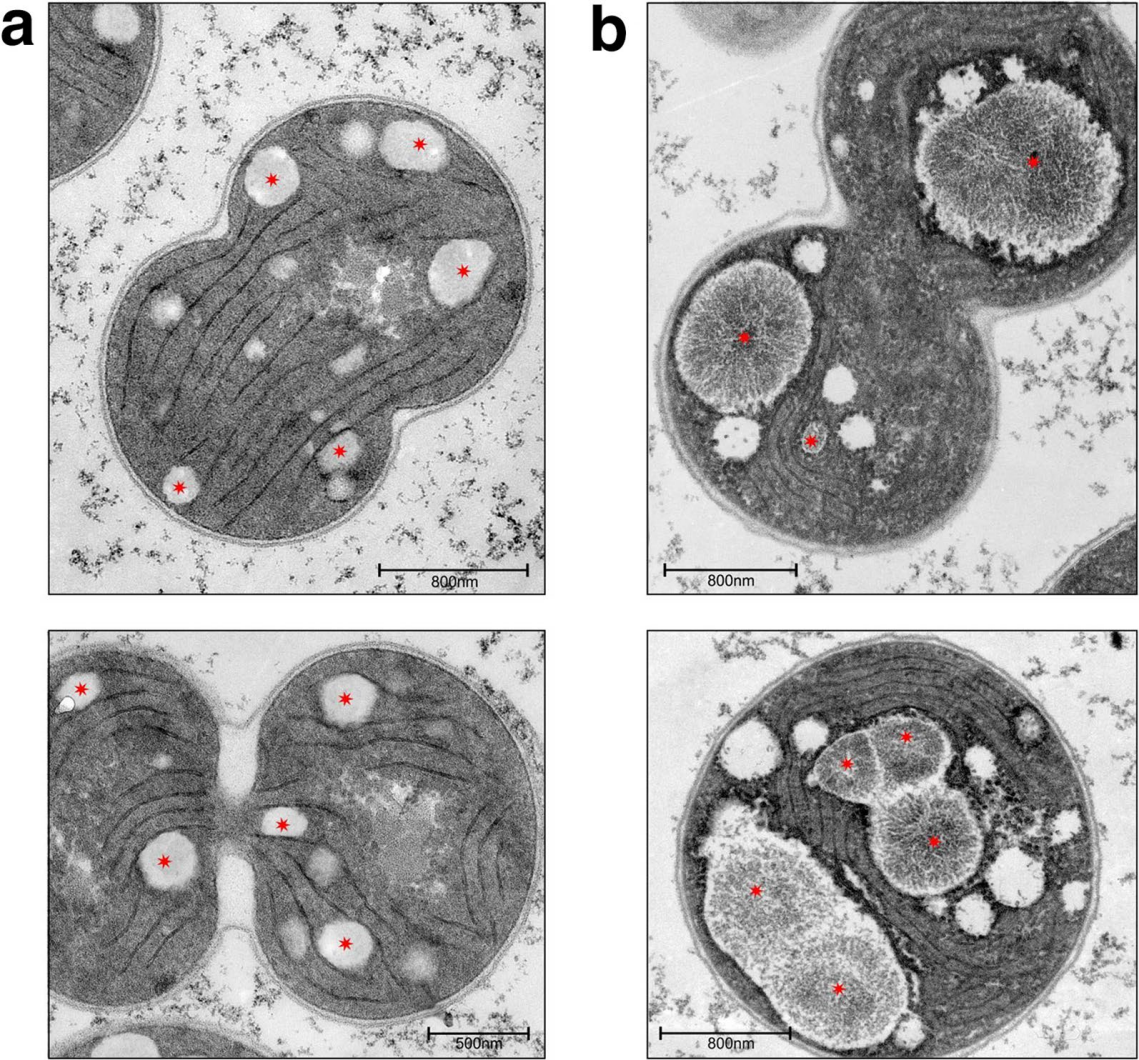
Transmissionelectron micrographs of ultrathin sections of phosphate-starved **a** *Synechocystis* sp. PCC 6803 wild-type and **b** strain BW86. *Red asterisks* indicate cyanophycin granules

The cytoplasm of exponentially growing wild-type cells stained light red due to the arginine content of cellular proteins. Dark red dots were visible only in very few cells. The intense color of these particles arises presumably from the high arginine content, and they would thus correspond to cyanophycin granules (Fig. 7a). Accordingly, the cytoplasm of cells of strain BW86 was clearly stained darker red due to the higher arginine content as well as more and larger cyanophycin granules, and the granules were heterogeneously distributed. During phosphate starvation, almost all cells, both from the wild-type and strain BW86, produced cyanophycin granules recognizable as dark red dots (Fig. 7b). The granules in strain BW86 were clearly larger than those in the wild-type; i.e., strain BW86 accumulated a few large cyanophycin granules compared to the small granules in the wild-type. We resolved the granule size in greater detail by examining phosphate-starved cells of wild-type and strain BW86 by transmission electron microscopy (Fig. 8). Cells of strain BW86 contained huge ovoid granules with a scar-like sub-structure (Fig. 8b). To our knowledge, these are the largest cyanophycin granules observed to date. In agreement with the above results, the cyanophycin granules of the wild-type were considerably smaller.

## Conclusions

This work demonstrated the possibility of metabolic engineering using the P_II_(I86N) variant of the P_II_ signal transduction protein to strongly increase arginine levels due to the P_II_ dependence of the key enzyme of arginine biosynthesis, NAGK. As a consequence, cells of the engineered strain, strain BW86, overproduced arginine and over-accumulated cyanophycin of high molecular weight. This direct link further supports our previous assumption that cyanophycin synthesis is primarily controlled by cellular arginine levels [23]. Taken together, the *Synechocystis* sp. strain described in this study, strain BW86, is the most potent cyanophycin producer described to date and is, therefore, a promising option for photoautotrophic production of arginine as well as cyanophycin. The large amount of cyanophycin of high molecular weight that can be produced with this strain opens the possibility of investigating novel applications of this polymer for biotechnological purposes.

In addition to the specific benefit of arginine and cyanophycin production obtained with the P_II_(I86N) variant, this study demonstrated the feasibility of using engineered variants of the P_II_ signaling protein for metabolic engineering in bacteria. In addition to arginine synthesis, P_II_ controls a multitude of other cellular activities in various autotrophic and heterotrophic bacteria. Therefore, metabolic pathways that are under control of this versatile regulatory protein could similarly be engineered.

## Methods

### Cultivation of bacteria

Standard cloning procedures were done in *E. coli* XL1-Blue (Stratagene) grown in Luria–Bertani medium at 37 °C with constant shaking at 300 rpm.

Cyanobacterial stains were grown photoautotrophically in BG-11 medium [38] containing nitrate or ammonium as nitrogen source and supplemented with 5 mM NaHCO_3_. Cultures were incubated in 50 or 200 ml medium in 100 or 500 ml Erlenmeyer flasks, respectively, at 28 °C with constant shaking at 120 rpm and illuminated with 50 µmol photons m^−2^ s^−1^. Larger cultures were grown in illuminated cylinders containing 800 ml medium supplemented with 5 mM NaHCO_3_, 5 mM TES/NaOH pH 8.2 (Roth). The cylinders were constantly aerated by bubbling 2 % CO_2_ in air through the liquid without additional shaking. Antibiotics were added to the media when required. Growth rates were monitored by measuring the optical density of the cultures at 750 nm.

Starvation conditions were induced by harvesting, washing, and transferring exponentially growing cells (OD_750_ 0.4–0.5) into BG-11 media lacking a specific nutrient, i.e., no nitrogen source for nitrogen starvation; K_2_HPO_4_ replaced by KCl for phosphate starvation; and K_2_HPO_4_ replaced by Na_2_HPO_4_ for potassium starvation. In the case of phosphate starvation, after 2 days cultures were diluted again in phosphate-free BG-11 medium to an OD_750_ of 0.15 to avoid entry into stationary phase before cellular phosphate reserves were exhausted.

### Construction of a P_II_(I86N)mutant

To construct a genomic P_II_(I86N) mutant of *Synechocystis* sp. PCC 6803, the P_II_-encoding gene *glnB* was genetically modified with the I86N mutation [Ile (5′ATC) at codon postion 86 to Asp (5′AAC)]. The entire cloning procedure is shown in Additional file 3: Figure S3. To incorporate the corresponding ATC to AAC mutation in the *glnB* gene, two amplicons of *glnB* were made using oligonucleotides P_II_(I86N)_rev (containing the ATC to AAC mutation) and P_II__prom_for as well as P_II__ter_rev (containing *Sac*I restriction site) and P_II_(I86N)_for. Both *glnB* amplicons were fused by fusion PCR resulting in the mutated *glnB* amplicon. The genomic region downstream of *glnB* was amplified using *slr0402*_rev (containing *Nde*I restriction site) and *slr0402*_for primers. The mutated *glnB* amplicon was fused with the *slr0402* fragment by fusion PCR and the resulting product was cloned in pJet 1.2 (Fermentas). Subsequently, a spectinomycin resistance cassette and a terminator sequence were inserted between the mutated *glnB* gene and the *slr0402* fragment for later selection. A spectinomycin resistance cassette was first amplified using oligonucleotides *spec*^*r*^_for (containing *Sac*I restriction site) and *spec*^*r*^_rev and was then fused with a terminator sequence that was, amplified using oligonucleotides *Ter*_rev (containing *Nde*I restriction site) and *Ter*_for. The product was inserted between the *SacI* and *NdeI* restriction sites of the *glnB*-*slr0402* fusion product, resulting in pJet P_II_(I86N). See Additional file 4: Table S1 for a list of all oligonucleotides used to generate constructs.

*Synechocystis* sp. PCC 6803 was transformed with pJet P_II_(I86N) via natural competence [39]; transformants were selected on BG-11 agar plates supplemented with 25 µg/ml spectinomycin. Transformants were screened for complete segregation via PCR using oligonucleotides P_II__prom_for and *slr0402*_for (Additional file 1: Figure S1).

### In vivo NAGK activity assay

Cells (20 ml of a 50-ml culture at an OD_750_ of 0.8) were rapidly harvested by centrifugation and resuspended in a buffer consisting of 50 mM Tris/HCl pH 7.4, 4 mM EDTA, 1 mM DTT, and 0.5 mM benzamidine. Cells were lysed using FastPrep^®^-24 (MP biomedical) with 0.1 mm glass beads at a speed of 6.0 m/s for 20 s five times. The lysate was separated into soluble and insoluble fractions by centrifugation at 25,000×*g* for 25 min at 4 °C. The protein concentration of the soluble fraction was determined using the Bradford assay [40]. NAGK activity of cell-free extract was measured according to Heinrich et al. [25] using 100 µg/ml protein for each measurement.

### Determination of P_II_ phosphorylation via western blotting

For non-denaturing electrophoretic separation of proteins, a native Gel was used according to Forchhammer et al. [30]. Per lane, 10 µg of crude protein extract was loaded; after electrophoretic separation, the gel was blotted onto a PVDF membrane [41]. The membrane was blocked with TBS blocking buffer (25 mM Tris/HCl pH 7.4, 75 mM NaCl) containing 1 % (v/v) Tween 20 overnight at 4 °C. The membrane was washed three times with TBS containing 0.1 % (v/v) Tween 20 (TBS-T) and afterwards incubated in TBS-T containing the anti-P_II_ antibody [20] for 1 h at ambient temperature. Unbound antibody was removed by washing three times with TBS-T. Anti-rabbit IgG secondary antibody conjugated to horseradish peroxidase (α-rabbit polyclonal goat antibody, Sigma-Aldrich) diluted 1:10,000 in TBS-T was applied to the membrane and incubated for 30 min at ambient temperature. Unbound antibodies were removed by three washes with TBS-T. Bound antibodies were visualized using the Lumi Light detection system (Roche Diagnostics). Luminograms were taken with the Gel Logic 1500 imaging system (Kodak) with the associated software.

### Metabolite extraction and quantification

For the extraction of metabolites, cells in 50 ml of culture at an OD_750_ of 0.8 were shock-cooled by mixing with crushed ice, rapidly harvested by centrifugation, and immediately frozen in liquid nitrogen. After freeze-drying, 5.0 mg lyophilized cells were homogenized with tungsten carbide beads in a Retsch Mill MM 200 (Retsch). Metabolites were extracted with 400 µl methanol, followed by a second extraction of the cells with 400 µl 20 % methanol with 0.1 % formic acid. The two supernatants were combined; solvents were removed using a vacuum concentrator. Metabolites were re-dissolved in 60 µl 20 % methanol with 0.1 % formic acid. A 5 µl aliquot was injected on a Waters UPLC/Synapt G2 LC/MS system equipped with a Waters Acquity 2.1 mm × 100 mm, 1.8 µm particle size HSS T3 reversed phase column. Metabolites were separated in a gradient from 20 % methanol with 0.1 % formic acid to 100 % methanol with 0.1 % formic acid in 10 min. The mass spectrometer was operated in ESI-positive and -negative modes with a scan range from *m/z* 50 to 2000 and a dwell time of 0.5 s. Data were evaluated using the MarkerLynx Software (Waters Cooperation, Milford, MA, USA) in combination with Simca-P (Umetrics AB Umea, Sweden). Compounds (based on formula and MS^E^ fragmentation pattern) that differed between the different samples were identified by principal component analysis (PCA) and Orthogonal projections to latent structures (OPLS)—discriminant analysis (DA). From the entire metabolome, this procedure therefore identifies only those compounds, whose abundance differs between the two analyzed samples.

### Cyanophycin extraction and quantification

Cyanophycin was extracted as described by Elbahloul et al. [42] with some modifications. Briefly, cells in 20–50 ml of culture at an OD_750_ of 0, 1–4 were harvested by centrifugation, resuspended in 100 % acetone, and incubated for 30 min with constant shaking at 1400 rpm. Cells were collected by centrifugation at 25,000×*g* for 15 min. The pellet was resuspended in 1.5 ml 0.1 M HCl and incubated for 1 h with constant shaking at 1400 rpm at 60 °C to solubilize cyanophycin. To remove debris, the sample was centrifuged at 25,000×*g* for 15 min. Cyanophycin in the clear supernatant was precipitated by adding 300 µl 1 M Tris/HCl pH 8.0 and incubating the mixture for 40 min at 4 °C. The mixture was then centrifuged at 25,000×*g* for 15 min at 4 °C. The supernatant was discarded, and the pelleted cyanophycin was dissolved in 500 µl 0.1 M HCl. Cyanophycin was quantified by determining arginine using the Sakaguchi reaction according to Messineo [43].

### Cyanophycin isolation and determination of molecular mass

To determine the molecular mass of native cyanophycin, we modified the procedure of Ziegler et al. [3] to avoid hydrolyzation of cyanophycin by acid extraction. Cells were harvested and washed three times in buffer consisting of 50 mM Tris/HCl pH 7.4, 150 mM NaCl, and 5 mM EDTA. The cell pellet was resuspended in B-Per™ buffer supplemented with 100 µg/ml lysozyme and 5 U/ml DNase I. Cells were lysed using FastPrep^®^-24 (MP biomedical) with 0.1 mm glass beads at a speed of 6.0 m/s for 20 s five times. The lysate was separated into soluble and insoluble fractions by centrifugation at 25,000×*g* for 25 min at 4 °C. The insoluble fraction was washed three times with the same buffer and resuspended in B-Per™ buffer supplemented with 200 µg/ml proteinase K, which does not degrade cyanophycin, and incubated at 50 °C overnight. Cyanophycin granules were collected by centrifugation at 25,000×*g* for 25 min at 4 °C and washed three times with water. Cyanophycin was quantified as described above. To determine the molecular mass, cyanopycin granules were solubilized in SDS-loading buffer and separated by SDS-PAGE on a 12 % polyacrylamide gel according to Sambrook and Russell [44].

### Microscopy and cyanophycin staining

Cyanophycin granules in bacterial cells were visualized microscopically using a newly developed staining method based on the Sakaguchi reaction. Cells of a 500 µl culture at an OD_750_ of 0.1–1.0 were collected via centrifugation and washed with 2.7 mM KCl, 1.5 mM KH_2_PO_4_, 137 mM NaCl, 8.1 mM Na_2_HPO_4_, pH 6.5 (PBS buffer). The cells were fixed by resuspending them in 500 µl PBS buffer containing 2.5 % (v/v) glutardialdehyde and incubated for 30 min at 4 °C. After fixation, the cells were washed with PBS and collected by centrifugation at 3000×*g* for 8 min at 4 °C. Then, cells were gently resuspended in 80 µl 5 M KOH and 10 µl of 1 % (w/v) 2,4-dichloro-1-naphthol dissolved in absolute ethanol was added; the mixture was incubated for 2 min at ambient temperature. Subsequently, 10 µl of 4–6 % (v/v) NaClO was added, and the mixture was incubated for 2 min. Finally, the cells were collected by centrifugation at 3000×*g* for 5 min, resuspended in 100 µl PBS buffer, and viewed under a Leica DM2500 microscope with a 100 ×/1.3 oil objective. Photographs were taken with a Leica DFC420C color camera.

### Transmissionelectron microscopy

Samples for transmissionelectron microscopy were prepared as described in Fiedler et al. [45]. Briefly, samples were fixed and post-fixed using glutaraldehyde and potassium permanganate. The samples were embedded in EPON, and ultrathin sections were stained with uranyl acetate and lead citrate. The samples were examined with a Philips Tecnai electron microscope at 80 kV.

## Additional files

10.1186/s12934-015-0384-4 Complete segregation of the mutation in the polyploidy *Synechocystis* sp. BW86. The replacement of all genomic copies of *glnB (ssl0707)* confirmed via PCR with isolated genomic DNA of *Synechocystis* sp. BW86, and, as controls, the wild-type *Synechocystis* PCC 6803 and the pJet 1.2 pJet P_II_(I86N) construct. Wild-type *glnB* produces a PCR fragment of approximately 1,300 bp, whereas the *glnB* (I86N) construct in both pJet 1.2 and strain BW86 produces a PCR fragment of approximately 2,600 bp with no observable wild-type background fragment.
10.1186/s12934-015-0384-4 Ammonium concentration in culture supernatants of the wild-type *Synechocystis* sp. PCC 6803 (WT) and strain BW86 grown in BG-11 medium with ammonium.
10.1186/s12934-015-0384-4 Cloning schemes for generation of the gene encoding P_II_(I86N) in *Synechocystis* sp. PCC 6803 showing all primer binding positions and restriction sites used in the construction. A) Spectinomycin resistance cassette (*spec* ^r^) fused with a terminator sequence (*Ter*). B) P_II_-encoding gene *glnB* and downstream open reading frame *slr0402*. C) Engineered construct encoding P_II_(I86N) with spectinomycin resistance cassette inserted downstream of the variant *glnB* gene.
10.1186/s12934-015-0384-4 Oligonucleotides used in this study.

## References

[1] Allen MM (1984). Cyanobacterial Cell Inclusions. Annu Rev Microbiol.

[2] Allen MM (1988). Cyanophycin—inclusions. Methods Enzymol.

[3] Ziegler K, Deutzmann R, Lockau W (2002). Cyanophycin synthetase-like enzymes of non-cyanobacterial eubacteria: characterization of the polymer produced by a recombinant synthetase of Desulfitobacterium hafniense. Z Naturforsch C..

[4] Krehenbrink M, Oppermann-Sanio FB, Steinbuchel A (2002). Evaluation of non-cyanobacterial genome sequences for occurrence of genes encoding proteins homologous to cyanophycin synthetase and cloning of an active cyanophycin synthetase from Acinetobacter sp. strain DSM 587. Arch Microbiol.

[5] Simon RD (1971). Cyanophycin granules from the blue-green alga *Anabaena cylindrica*: a reserve material consisting of copolymers of aspartic acid and arginine. Proc Natl Acad Sci USA..

[6] Simon RD (1976). The biosynthesis of multi-l-arginyl-poly(l-aspartic acid) in the filamentous cyanobacterium *Anabaena cylindrica*. Biochim Biophys Acta.

[7] Aboulmagd E, Oppermann-Sanio FB, Steinbuchel A (2000). Molecular characterization of the cyanophycin synthetase from Synechocystis sp. strain PCC6308. Arch Microbiol.

[8] Berg H, Ziegler K, Piotukh K, Baier K, Lockau W, Volkmer-Engert R (2000). Biosynthesis of the cyanobacterial reserve polymer multi-l-arginyl-poly-l-aspartic acid (cyanophycin): mechanism of the cyanophycin synthetase reaction studied with synthetic primers. Eur J Biochem.

[9] Allen MM, Weathers PJ (1980). Structure and composition of cyanophycin granules in the cyanobacterium Aphanocapsa 6308. J Bacteriol.

[10] Hegler R (1901). Untersuchungen üiber die Organisation der Phycochromaceenzellen. Jb wiss Bot.

[11] Simon RD (1973). Measurement of the cyanophycin granule polypeptide contained in the blue-green alga *Anabaena cylindrica*. J Bacteriol.

[12] Obst M, Steinbuchel A (2004). Microbial degradation of poly(amino acid)s. Biomacromolecules.

[13] Simon RD (1973). The effect of chloramphenicol on the production of cyanophycin granule polypeptide in the blue green alga *Anabaena cylindrica*. Arch Mikrobiol..

[14] Allen MM, Hutchison F (1980). Nitrogen limitation and recovery in the cyanobacterium aphanocapsa-6308. Arch Microbiol.

[15] Ziegler K, Stephan DP, Pistorius EK, Ruppel HG, Lockau W (2001). A mutant of the cyanobacterium *Anabaena variabilis* ATCC 29413 lacking cyanophycin synthetase: growth properties and ultrastructural aspects. FEMS Microbiol Lett.

[16] Forchhammer K (2004). Global carbon/nitrogen control by PII signal transduction in cyanobacteria: from signals to targets. FEMS Microbiol Rev.

[17] Zeth K, Fokina O, Forchhammer K (2014). Structural basis and target-specific modulation of ADP sensing by the *Synechococcus elongatus* PII signaling protein. J Biol Chem.

[18] Forchhammer K (2008). P(II) signal transducers: novel functional and structural insights. Trends Microbiol.

[19] Fokina O, Chellamuthu VR, Forchhammer K, Zeth K (2010). Mechanism of 2-oxoglutarate signaling by the *Synechococcus elongatus* PII signal transduction protein. Proc Natl Acad Sci USA..

[20] Forchhammer K, Hedler A (1997). Phosphoprotein PII from cyanobacteria–analysis of functional conservation with the PII signal-transduction protein from *Escherichia coli*. Eur J Biochem.

[21] Espinosa J, Forchhammer K, Burillo S, Contreras A (2006). Interaction network in cyanobacterial nitrogen regulation: PipX, a protein that interacts in a 2-oxoglutarate dependent manner with PII and NtcA. Mol Microbiol.

[22] Osanai T, Sato S, Tabata S, Tanaka K (2005). Identification of PamA as a PII-binding membrane protein important in nitrogen-related and sugar-catabolic gene expression in Synechocystis sp. PCC 6803. J Biol Chem.

[23] Maheswaran M, Ziegler K, Lockau W, Hagemann M, Forchhammer K (2006). PII-regulated arginine synthesis controls accumulation of cyanophycin in Synechocystis sp. strain PCC 6803. J Bacteriol.

[24] Caldovic L, Tuchman M (2003). *N*-acetylglutamate and its changing role through evolution. Biochem J.

[25] Heinrich A, Maheswaran M, Ruppert U, Forchhammer K (2004). The *Synechococcus elongatus* P signal transduction protein controls arginine synthesis by complex formation with *N*-acetyl-l-glutamate kinase. Mol Microbiol.

[26] Fokina O, Chellamuthu VR, Zeth K, Forchhammer K (2010). A novel signal transduction protein P(II) variant from *Synechococcus elongatus* PCC 7942 indicates a two-step process for NAGK-P(II) complex formation. J Mol Biol.

[27] Zeth K, Fokina O, Forchhammer K (2012). An engineered PII protein variant that senses a novel ligand: atomic resolution structure of the complex with citrate. Acta Crystallogr D..

[28] Forchhammer K, Tandeau de Marsac N (1995). Phosphorylation of the PII protein (glnB gene product) in the cyanobacterium Synechococcus sp. strain PCC 7942: analysis of in vitro kinase activity. J Bacteriol.

[29] Krasikov V, AguirrevonWobeser E, Dekker HL, Huisman J, Matthijs HC (2012). Time-series resolution of gradual nitrogen starvation and its impact on photosynthesis in the cyanobacterium Synechocystis PCC 6803. Physiol Plant..

[30] Forchhammer K, Tandeau de Marsac N (1994). The PII protein in the cyanobacterium Synechococcus sp. strain PCC 7942 is modified by serine phosphorylation and signals the cellular N-status. J Bacteriol.

[31] Allen MM, Hutchison F, Weathers PJ (1980). Cyanophycin granule polypeptide formation and degradation in the cyanobacterium Aphanocapsa 6308. J Bacteriol.

[32] Grillo JF, Gibson J (1979). Regulation of phosphate accumulation in the unicellular cyanobacterium Synechococcus. J Bacteriol.

[33] Alahari A, Apte SK (1998). Pleiotropic effects of potassium deficiency in a heterocystous, nitrogen-fixing cyanobacterium, *Anabaena torulosa*. Microbiol-Uk..

[34] Shin JH, Lee SY (2014). Metabolic engineering of microorganisms for the production of l-arginine and its derivatives. Microb Cell Fact.

[35] Steinle A, Oppermann-Sanio FB, Reichelt R, Steinbuchel A (2008). Synthesis and accumulation of cyanophycin in transgenic strains of *Saccharomyces cerevisiae*. Appl Environ Microbiol.

[36] Frey KM, Oppermann-Sanio FB, Schmidt H, Steinbuchel A (2002). Technical-scale production of cyanophycin with recombinant strains of *Escherichia coli*. Appl Environ Microbiol.

[37] Deitch AD (1961). An improved Sakaguchi reaction for microspectrophotometric use. J Histochem Cytochem.

[38] Rippka R, Deruelles J, Waterbury JB, Herdman M, Stanier RY (1979). Generic assignments, strain histories and properties of pure cultures of cyanobacteria. J Gen Microbiol.

[39] Grigorieva G, Shestakov S (1982). Transformation in the cyanobacterium *Synechocystis* sp. 6803. FEMS Microbiol Lett.

[40] Bradford MM (1976). A rapid and sensitive method for the quantitation of microgram quantities of protein utilizing the principle of protein-dye binding. Anal Biochem.

[41] Towbin H, Staehelin T, Gordon J (1979). Electrophoretic transfer of proteins from polyacrylamide gels to nitrocellulose sheets: procedure and some applications. Proc Natl Acad Sci USA..

[42] Elbahloul Y, Krehenbrink M, Reichelt R, Steinbuchel A (2005). Physiological conditions conducive to high cyanophycin content in biomass of *Acinetobacter calcoaceticus* strain ADP1. Appl Environ Microbiol.

[43] Messineo L (1966). Modification of Sakaguchi reaction—spectrophotometric determination of arginine in proteins without previous hydrolysis. Arch Biochem Biophys.

[44] Sambrook J, Russell D (2001). Molecular cloning: a laboratory manual.

[45] Fiedler G, Arnold M, Hannus S, Maldener I (1998). The DevBCA exporter is essential for envelope formation in heterocysts of the cyanobacterium Anabaena sp. strain PCC 7120. Mol Microbiol.

