# Supplementary figures and images for: Metabolic pathway engineering using the central signal processor PII
Source: Microb Cell Fact. 2015 Nov 25;14:192. doi: 10.1186/s12934-015-0384-4 (PMC4660640; doi:10.1186/s12934-015-0384-4)

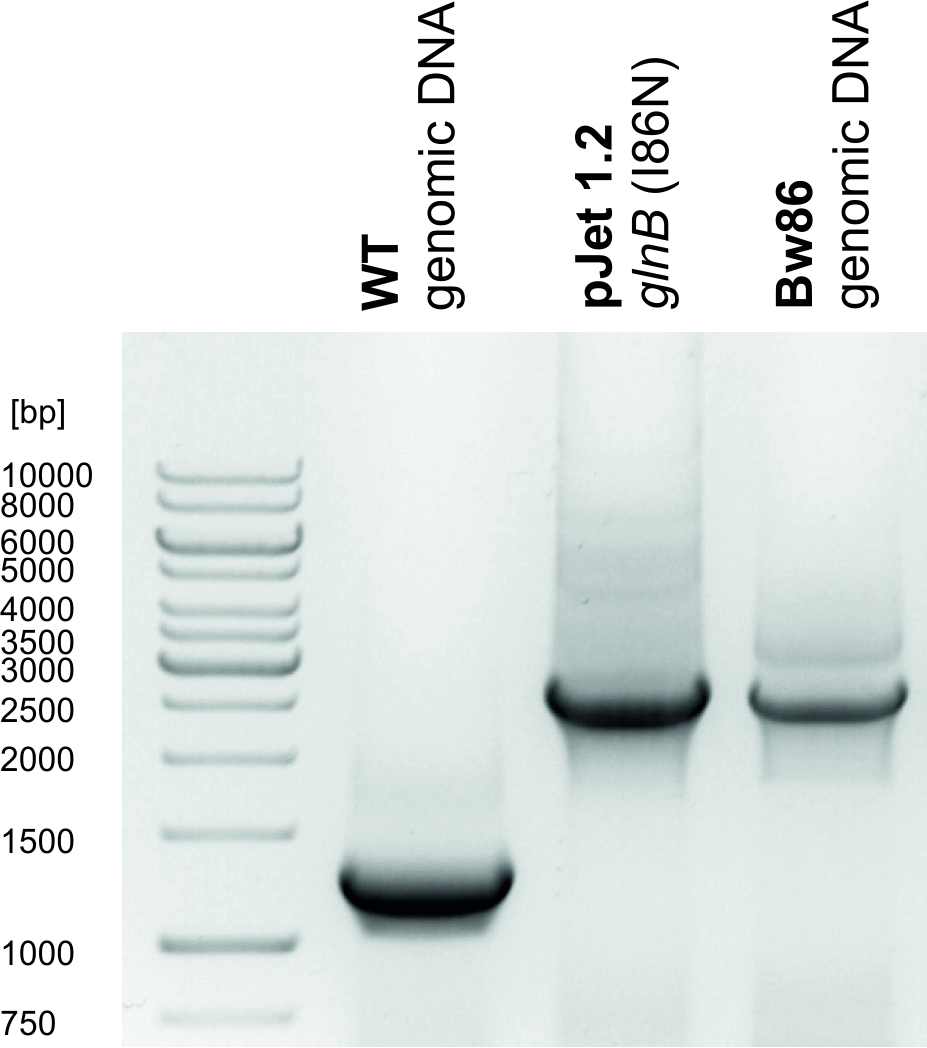

Supplement: Supplementary file 1 — 10.1186/s12934-015-0384-4 Complete segregation of the mutation in the polyploidy Synechocystis sp. BW86. The replacement of all genomic copies of glnB (ssl0707) confirmed via PCR with isolated genomic DNA of Synechocystis sp. BW86, and, as controls, the wild-type Synechocystis PCC 6803 and the pJet 1.2 pJet PII(I86N) construct. Wild-type glnB produces a PCR fragment of approximately 1,300 bp, whereas the glnB (I86N) construct in both pJet 1.2 and strain BW86 produces a PCR fragment of approximately 2,600 bp with no observable wild-type background fragment. [file 12934_2015_384_MOESM1_ESM.jpg]

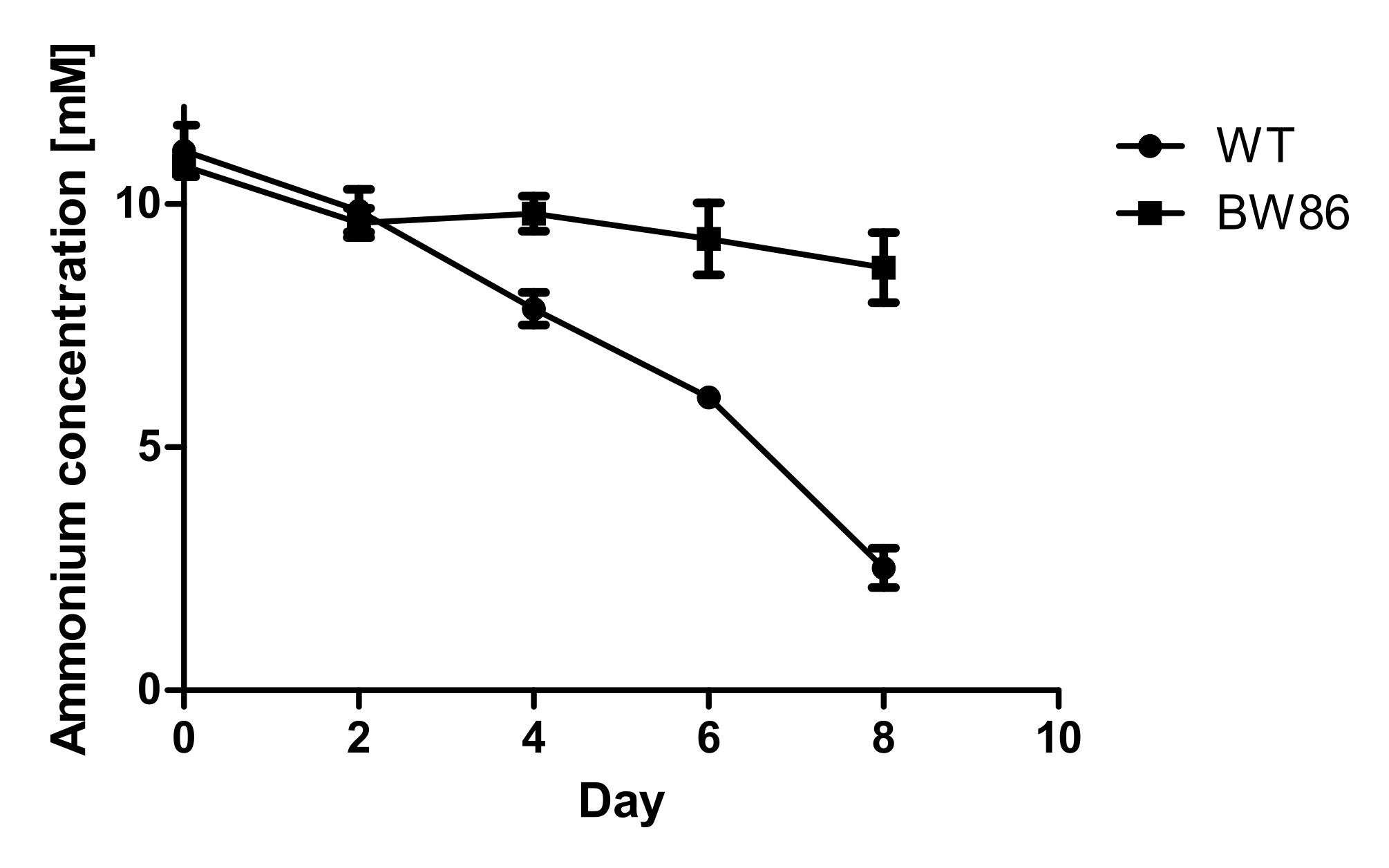

Supplement: Supplementary file 2 — 10.1186/s12934-015-0384-4 Ammonium concentration in culture supernatants of the wild-type Synechocystis sp. PCC 6803 (WT) and strain BW86 grown in BG-11 medium with ammonium. [file 12934_2015_384_MOESM2_ESM.jpg]

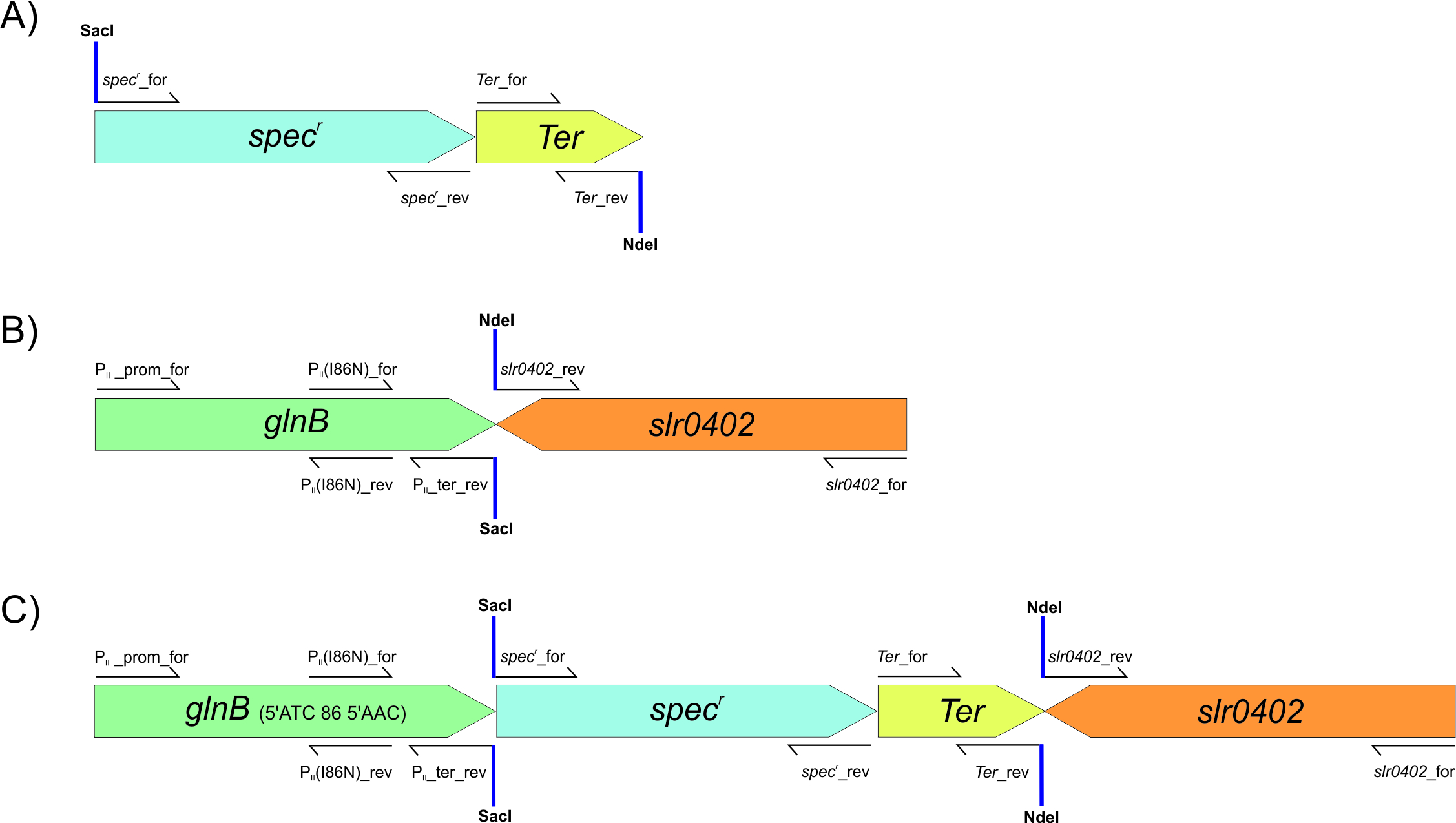

Supplement: Supplementary file 3 — 10.1186/s12934-015-0384-4 Cloning schemes for generation of the gene encoding PII(I86N) in Synechocystis sp. PCC 6803 showing all primer binding positions and restriction sites used in the construction. A) Spectinomycin resistance cassette (spec r) fused with a terminator sequence (Ter). B) PII-encoding gene glnB and downstream open reading frame slr0402. C) Engineered construct encoding PII(I86N) with spectinomycin resistance cassette inserted downstream of the variant glnB gene. [file 12934_2015_384_MOESM3_ESM.jpg]
