# Supplementary material for: Metabolic pathway engineering using the central signal processor PII
Source: Microb Cell Fact. 2015 Nov 25;14:192. doi: 10.1186/s12934-015-0384-4 (PMC4660640; doi:10.1186/s12934-015-0384-4)
Supplement: Supplementary file 4 — 10.1186/s12934-015-0384-4 Oligonucleotides used in this study. [file 12934_2015_384_MOESM4_ESM.docx]

| **Primer** | **Sequence** |
| --- | --- |
|  |  |
| P_II_(I86N)_for | 5´-CACCGGGGAAAACGGTGACGGTAAAATC-3´ |
| P_II_(I86N)_rev | 5´-GATTTTACCGTCACCGTTTTCCCCGGTG-3´ |
| P_II__prom_for | 5´-CTAGGACTCCCTGGTCAAGGGTTCACC-3´ |
| P_II__ter_rev | 5´-CCAACATTAACGCATATGGATCGAGCTCGCAACCG-GAAAGCTAATCTCAAAAAG-3´ |
| *slr0402*_for | 5´-GCCTACTTTAATTGTCGCCACGGGTAAC-3´ |
| *slr0402*_rev | 5´-GAGCTCGATCCATATGCGTTAATGTTGGGCAGATTGAGACAAGTCTC-3´ |
| *spec^r^*_for | 5'-GATCGAGCTCTTGACCGAACGCAGCGGTGGTAACG-3' |
| *spec^r^*_rev | 5'-CAAATTCTGTTTTATCAGACCGCTTCTGCTTATTTGCCGACTACCTT-GGTGATCTCG-3' |
| *Ter*_for | 5'-GCAGAAGCGGTCTGATAAAACAGAATTTG-3' |
| *Ter*_rev | 5'-GATCCATATGGAGTTTGTAGAAACGCAAAAAGGCCATC-3' |
|  |  |
